# 12α-Hy­droxy-3,27-dioxooleanano-28,13-lactone

**DOI:** 10.1107/S1600536812017783

**Published:** 2012-05-02

**Authors:** Jun-yi Hu, Gang-gang Wu, Ying-qian Xu, Guo-yong Xiao, Peng Lei

**Affiliations:** a Center of Separation Technology, School of Chemical Engineering, University of Science and Technology Liaoning, Anshan 114051, People’s Republic of China

## Abstract

There are two independent mol­ecules in the asymmetric unit of the title compound, C_30_H_44_O_5_. They comprise a triterpenoid skeleton of five six-membered rings and a five-membered lactone ring. The five six-membered rings are all *trans*-fused. In both independent mol­ecules the *D* rings adopt a slightly distorted half-chair conformation due the presence of the lactone ring while the other four six-membered rings all adopt chair conformations. The characteristic carbon–carbon double bond of the oleanoic skeleton is absent. Inter­molecular O—H⋯O hydrogen bonds between the hy­droxy and carbonyl groups occur in the crystal structure.

## Related literature

For the pharmacological properties of penta­cyclic triterpene acids, see: Gene *et al.* (1996 ▶); Hu *et al.* (2009 ▶); Xu *et al.* (2007 ▶).
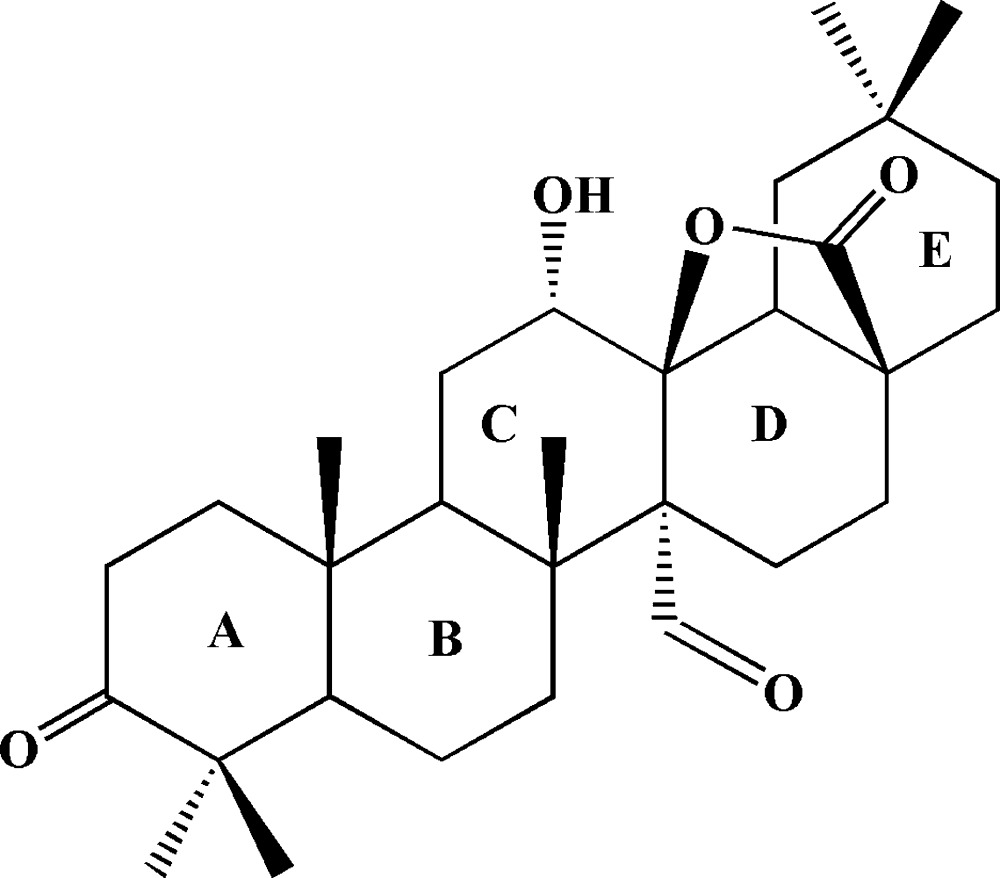


## Experimental

### 

#### Crystal data

C_30_H_44_O_5_
*M* *_r_* = 484.65
Orthorhombic, 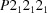

*a* = 12.4457 (3) Å
*b* = 15.5804 (4) Å
*c* = 27.1710 (8) Å
*V* = 5268.7 (2) Å^3^
*Z* = 8
Mo *K*α radiation
μ = 0.08 mm^−1^
*T* = 113 K
0.32 × 0.30 × 0.26 mm

#### Data collection

Rigaku Saturn CCD area-detector diffractometer
Absorption correction: multi-scan (*CrystalClear*; Rigaku, 2007 ▶) *T*_min_ = 0.975, *T*_max_ = 0.979
46805 measured reflections
6391 independent reflections
6106 reflections with *I* > 2σ(*I*)
*R*_int_ = 0.054

#### Refinement

*R*[*F* ^2^ > 2σ(*F* ^2^)] = 0.041
*wR*(*F* ^2^) = 0.095
*S* = 1.10
6391 reflections
649 parameters
H atoms treated by a mixture of independent and constrained refinement
Δρ_max_ = 0.18 e Å^−3^
Δρ_min_ = −0.23 e Å^−3^

### 

Data collection: *CrystalClear* (Rigaku, 2007 ▶); cell refinement: *CrystalClear*; data reduction: *CrystalClear*; program(s) used to solve structure: *SHELXS97* (Sheldrick, 2008 ▶); program(s) used to refine structure: *SHELXL97* (Sheldrick, 2008 ▶); molecular graphics: *SHELXTL* (Sheldrick, 2008 ▶); software used to prepare material for publication: *CrystalStructure* (Rigaku, 2007 ▶).

## Supplementary Material

Crystal structure: contains datablock(s) global, I. DOI: 10.1107/S1600536812017783/kp2404sup1.cif

Structure factors: contains datablock(s) I. DOI: 10.1107/S1600536812017783/kp2404Isup2.hkl

Additional supplementary materials:  crystallographic information; 3D view; checkCIF report

## Figures and Tables

**Table 1:** Hydrogen-bond geometry (Å, °)

*D*—H⋯*A*	*D*—H	H⋯*A*	*D*⋯*A*	*D*—H⋯*A*
O2—H2⋯O4^i^	0.86 (3)	1.94 (3)	2.782 (2)	167 (3)
O7—H7⋯O6^ii^	0.90 (3)	1.91 (3)	2.803 (2)	172 (2)

Symmetry codes: (i) 
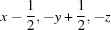
; (ii) 
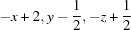
.

**Fig. 1.**
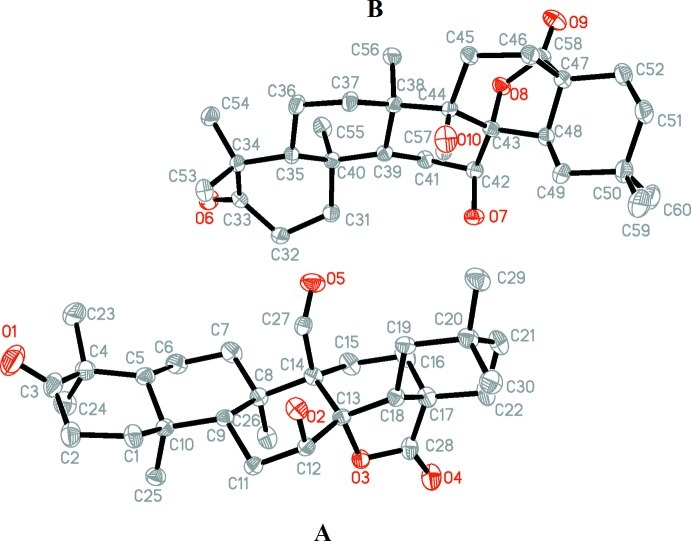
The two molecules of an asymmetric unit of (I) with the atom-labelling scheme. Displacement ellipsoids are drawn at the 60% probability level and H atoms have been omitted for clarity.

## supplementary crystallographic information

### Comment

Pentacyclic triterpene acids such as oleanolic acid, crataegolic acid and ursolic acid and their glycosides are widely distributed in the plant kingdom. They exhibit a variety of pharmacological properties such as analgesic (Gene *et al.*, 1996), cytotoxic (Hu *et al.*, 2009), and immunosuppressive activities (Xu *et al.*, 2007). Recently, our laboratories have disclosed the design and synthesis of a novel series of oleanolic acid derivatives. Herewith, we report the synthesis and crystal structure of the title compound. The title compound crystallizes with two molecules per asymmetric unit (Fig. 1). Each molecule contains a five-membered lactone ring and five *trans*-fused cyclohexane rings *via* A(C1—C5, C10), B(C5—C10), C(C8, C9, C11—C14), D(C13—C18) and E(C17—C22) in the molecule A, respectively, and A'(C31—C35, C40), B'(C35—C40), C'(C38, C39, C41—C44), D'(C43—C48) and E'(C47—C52) in the molecule B, respectively. The characteristical carbon double bond of oleanoic skeleton is absent in the title compound. Ring A(A'), B(B'), C(C') and E(E') adopt chair conformations, while ring D(D') adopts a slightly distorted half-chair conformation as a result of the formation of the lactone ring. The hydroxy groups attached to atom C12 and C42 are equatorial. The intermolecular O—H···O hydrogen bonds were observed between the hydroxy groups and carbonyl groups (Table 1) which stablize the crystal structure.

### Experimental

Commercially available oleanolic acid and Jones reagent in dry CH_2_Cl_2_/acetone (1/1) was stirred at 278 K for 1 h to give 3-oxoolean-12-en-28-oic acid. Reaction of 3-oxoolean-12-en-28-oic acid with O_3_ in CH_2_Cl_2_/MeOH (1:1) at 223 K gave 12-hydroxy-3-oxooleanano -28,13-lactone. A stream of NOCl was introduced to an ice-cold solution of 12-hydroxy-3-oxooleanano-28,13-lactone in pyridine at 233 K over 30 min to give lactone nitrite. A solution of lactone nitrite in acetone was irradiated by high pressure Hg lamp through a Pyrex filter under nitrogen atmosphere for 1 h to give oxime. To a solution of oxime in dioxane and AcOH, aqueous TiCl_3_ was added under ice, keeping temperature of 299 K by cooling, for 4 h to give imine. To imine in dioxane and AcOH, an aqueous solution of NaNO_2_ was added for 1 h to obtain the title compound. Crystals suitable for X-ray structure analysis were obtained by slow evaporation of a solution in methanol at room temperature.

### Refinement

H atoms of the hydroxy group were located in a difference density map and refined freely. Other H atoms were positioned geometrically and refined as riding (C—H = 0.95–1.00 Å) and allowed to ride on their parent atoms, with *U*_iso_(H) = 1.2*U*_eq_ or 1.5*U*_eq_ (parent). The absolute configuration could not be established because of the absence of significant anomalous effects. Friedel pairs were merged for the final cycles of refinement.

### Crystal data

**Table:**

C_30_H_44_O_5_	*F*(000) = 2112
*M**_r_* = 484.65	*D*_x_ = 1.222 Mg m^−^^3^
Orthorhombic, *P*2_1_2_1_2_1_	Mo *K*α radiation, λ = 0.71073 Å
Hall symbol: P 2ac 2ab	Cell parameters from 11606 reflections
*a* = 12.4457 (3) Å	θ = 1.5–27.1°
*b* = 15.5804 (4) Å	µ = 0.08 mm^−^^1^
*c* = 27.1710 (8) Å	*T* = 113 K
*V* = 5268.7 (2) Å^3^	Block, colourless
*Z* = 8	0.32 × 0.30 × 0.26 mm

### Data collection

**Table:**

Rigaku Saturn CCD area-detector diffractometer	6391 independent reflections
Radiation source: rotating anode	6106 reflections with *I* > 2σ(*I*)
Multilayer monochromator	*R*_int_ = 0.054
Detector resolution: 7.31 pixels mm^-1^	θ_max_ = 27.1°, θ_min_ = 1.5°
φ and ω scans	*h* = −15→15
Absorption correction: multi-scan (*CrystalClear*; Rigaku, 2007)	*k* = −19→19
*T*_min_ = 0.975, *T*_max_ = 0.979	*l* = −34→32
46805 measured reflections	

### Refinement

**Table:**

Refinement on *F*^2^	Primary atom site location: structure-invariant direct methods
Least-squares matrix: full	Secondary atom site location: difference Fourier map
*R*[*F*^2^ > 2σ(*F*^2^)] = 0.041	Hydrogen site location: inferred from neighbouring sites
*wR*(*F*^2^) = 0.095	H atoms treated by a mixture of independent and constrained refinement
*S* = 1.10	*w* = 1/[σ^2^(*F*_o_^2^) + (0.0492*P*)^2^ + 0.7562*P*] where *P* = (*F*_o_^2^ + 2*F*_c_^2^)/3
6391 reflections	(Δ/σ)_max_ < 0.001
649 parameters	Δρ_max_ = 0.18 e Å^−^^3^
0 restraints	Δρ_min_ = −0.23 e Å^−^^3^

### Special details

**Table:**

Geometry. All e.s.d.'s (except the e.s.d. in the dihedral angle between two l.s. planes) are estimated using the full covariance matrix. The cell e.s.d.'s are taken into account individually in the estimation of e.s.d.'s in distances, angles and torsion angles; correlations between e.s.d.'s in cell parameters are only used when they are defined by crystal symmetry. An approximate (isotropic) treatment of cell e.s.d.'s is used for estimating e.s.d.'s involving l.s. planes.
Refinement. Refinement of *F*^2^ against ALL reflections. The weighted *R*-factor *wR* and goodness of fit *S* are based on *F*^2^, conventional *R*-factors *R* are based on *F*, with *F* set to zero for negative *F*^2^. The threshold expression of *F*^2^ > σ(*F*^2^) is used only for calculating *R*-factors(gt) *etc*. and is not relevant to the choice of reflections for refinement. *R*-factors based on *F*^2^ are statistically about twice as large as those based on *F*, and *R*- factors based on ALL data will be even larger.

### Fractional atomic coordinates and isotropic or equivalent isotropic displacement parameters (Å^2^)

**Table:**

	*x*	*y*	*z*	*U*_iso_*/*U*_eq_	
O1	0.58097 (18)	0.75511 (12)	0.07251 (8)	0.0470 (5)	
O2	0.58149 (12)	0.28995 (10)	0.04638 (6)	0.0201 (3)	
H2	0.537 (2)	0.3181 (17)	0.0285 (10)	0.030*	
O3	0.85004 (12)	0.21425 (9)	0.02152 (5)	0.0187 (3)	
O4	0.96348 (12)	0.10366 (10)	0.01557 (6)	0.0285 (4)	
O5	0.74482 (14)	0.30826 (11)	0.17851 (6)	0.0293 (4)	
O6	0.94971 (13)	0.54315 (10)	0.28292 (6)	0.0263 (4)	
O7	0.85259 (13)	0.05664 (10)	0.26587 (5)	0.0204 (3)	
H7	0.916 (2)	0.0577 (16)	0.2496 (10)	0.031*	
O8	0.81857 (12)	0.02374 (9)	0.39691 (5)	0.0188 (3)	
O9	0.78339 (14)	−0.06106 (11)	0.46169 (6)	0.0287 (4)	
O10	0.52822 (12)	0.08913 (10)	0.29367 (6)	0.0258 (4)	
C1	0.62798 (17)	0.55293 (14)	0.01435 (9)	0.0225 (5)	
H1A	0.6143	0.5266	−0.0182	0.027*	
H1B	0.5757	0.5283	0.0379	0.027*	
C2	0.6088 (2)	0.65032 (14)	0.01065 (9)	0.0276 (5)	
H2A	0.6510	0.6732	−0.0173	0.033*	
H2B	0.5319	0.6607	0.0035	0.033*	
C3	0.6392 (2)	0.69874 (14)	0.05659 (10)	0.0301 (6)	
C4	0.7475 (2)	0.67756 (14)	0.08065 (9)	0.0284 (5)	
C5	0.76449 (19)	0.57790 (14)	0.08003 (8)	0.0217 (5)	
H5	0.7090	0.5553	0.1033	0.026*	
C6	0.8717 (2)	0.54981 (14)	0.10253 (9)	0.0256 (5)	
H6A	0.8875	0.5851	0.1319	0.031*	
H6B	0.9302	0.5585	0.0784	0.031*	
C7	0.86607 (19)	0.45489 (14)	0.11710 (8)	0.0245 (5)	
H7A	0.8095	0.4475	0.1424	0.029*	
H7B	0.9354	0.4379	0.1320	0.029*	
C8	0.84185 (17)	0.39469 (13)	0.07335 (8)	0.0179 (4)	
C9	0.74243 (17)	0.43067 (12)	0.04433 (7)	0.0162 (4)	
H9	0.6802	0.4233	0.0672	0.019*	
C10	0.74303 (17)	0.52904 (13)	0.03119 (7)	0.0177 (4)	
C11	0.71794 (16)	0.37080 (13)	0.00072 (7)	0.0170 (4)	
H11A	0.6616	0.3968	−0.0203	0.020*	
H11B	0.7834	0.3633	−0.0195	0.020*	
C12	0.67976 (17)	0.28344 (13)	0.01944 (8)	0.0165 (4)	
H12	0.6681	0.2448	−0.0094	0.020*	
C13	0.76031 (16)	0.24126 (13)	0.05412 (7)	0.0157 (4)	
C14	0.81068 (16)	0.30106 (13)	0.09439 (8)	0.0172 (4)	
C15	0.90918 (18)	0.25408 (14)	0.11685 (9)	0.0238 (5)	
H15A	0.9707	0.2596	0.0940	0.029*	
H15B	0.9292	0.2826	0.1481	0.029*	
C16	0.88884 (18)	0.15814 (15)	0.12701 (9)	0.0249 (5)	
H16A	0.8434	0.1526	0.1568	0.030*	
H16B	0.9583	0.1297	0.1339	0.030*	
C17	0.83308 (17)	0.11138 (14)	0.08360 (8)	0.0201 (4)	
C18	0.72330 (16)	0.15241 (13)	0.07333 (8)	0.0166 (4)	
H18	0.6938	0.1215	0.0440	0.020*	
C19	0.64336 (17)	0.13627 (13)	0.11489 (8)	0.0179 (4)	
H19A	0.5729	0.1616	0.1062	0.021*	
H19B	0.6691	0.1642	0.1454	0.021*	
C20	0.63048 (17)	0.03816 (14)	0.12369 (8)	0.0204 (4)	
C21	0.74144 (19)	−0.00440 (15)	0.13175 (9)	0.0252 (5)	
H21A	0.7711	0.0157	0.1635	0.030*	
H21B	0.7314	−0.0673	0.1342	0.030*	
C22	0.82348 (19)	0.01443 (14)	0.09095 (9)	0.0263 (5)	
H22A	0.8943	−0.0097	0.1001	0.032*	
H22B	0.8001	−0.0130	0.0599	0.032*	
C23	0.7453 (3)	0.70955 (16)	0.13443 (10)	0.0451 (8)	
H23A	0.7281	0.7709	0.1350	0.068*	
H23B	0.8159	0.7003	0.1495	0.068*	
H23C	0.6906	0.6778	0.1529	0.068*	
C24	0.8352 (2)	0.72879 (16)	0.05322 (10)	0.0329 (6)	
H24A	0.8312	0.7162	0.0179	0.049*	
H24B	0.9061	0.7123	0.0658	0.049*	
H24C	0.8239	0.7903	0.0586	0.049*	
C25	0.82047 (17)	0.55160 (14)	−0.01128 (8)	0.0211 (4)	
H25A	0.8205	0.5050	−0.0355	0.032*	
H25B	0.8932	0.5593	0.0018	0.032*	
H25C	0.7968	0.6049	−0.0271	0.032*	
C26	0.94445 (17)	0.39040 (15)	0.04132 (9)	0.0235 (5)	
H26A	0.9582	0.4469	0.0267	0.035*	
H26B	0.9344	0.3480	0.0151	0.035*	
H26C	1.0057	0.3737	0.0619	0.035*	
C27	0.72566 (19)	0.31387 (13)	0.13495 (8)	0.0201 (4)	
H27	0.6542	0.3269	0.1252	0.024*	
C28	0.89023 (17)	0.13821 (14)	0.03740 (9)	0.0211 (5)	
C29	0.56350 (19)	0.02642 (16)	0.17039 (9)	0.0281 (5)	
H29A	0.4922	0.0516	0.1655	0.042*	
H29B	0.5993	0.0550	0.1980	0.042*	
H29C	0.5561	−0.0349	0.1776	0.042*	
C30	0.5710 (2)	−0.00436 (15)	0.08081 (9)	0.0256 (5)	
H30A	0.5601	−0.0654	0.0881	0.038*	
H30B	0.6136	0.0015	0.0507	0.038*	
H30C	0.5011	0.0235	0.0762	0.038*	
C31	0.93563 (18)	0.31539 (14)	0.27350 (8)	0.0207 (5)	
H31A	1.0092	0.3015	0.2848	0.025*	
H31B	0.9139	0.2707	0.2496	0.025*	
C32	0.93773 (19)	0.40317 (13)	0.24707 (8)	0.0217 (5)	
H32A	1.0123	0.4154	0.2365	0.026*	
H32B	0.8928	0.3994	0.2171	0.026*	
C33	0.89835 (17)	0.47677 (13)	0.27798 (8)	0.0194 (4)	
C34	0.78608 (17)	0.46634 (13)	0.29976 (8)	0.0189 (4)	
C35	0.75950 (16)	0.36869 (13)	0.30709 (8)	0.0166 (4)	
H35	0.7315	0.3483	0.2746	0.020*	
C36	0.66828 (17)	0.35359 (13)	0.34393 (8)	0.0209 (5)	
H36A	0.6068	0.3913	0.3356	0.025*	
H36B	0.6932	0.3687	0.3774	0.025*	
C37	0.63149 (17)	0.25966 (13)	0.34323 (8)	0.0196 (4)	
H37A	0.6046	0.2454	0.3099	0.024*	
H37B	0.5712	0.2524	0.3666	0.024*	
C38	0.72235 (17)	0.19660 (13)	0.35705 (7)	0.0164 (4)	
C39	0.81945 (16)	0.21624 (13)	0.32225 (7)	0.0154 (4)	
H39	0.7928	0.2017	0.2886	0.018*	
C40	0.85859 (16)	0.31201 (13)	0.31817 (8)	0.0164 (4)	
C41	0.91204 (16)	0.15334 (13)	0.33136 (8)	0.0179 (4)	
H41A	0.9752	0.1705	0.3115	0.021*	
H41B	0.9328	0.1549	0.3665	0.021*	
C42	0.87781 (16)	0.06313 (13)	0.31740 (8)	0.0170 (4)	
H42	0.9383	0.0229	0.3249	0.020*	
C43	0.77906 (16)	0.03404 (13)	0.34572 (7)	0.0159 (4)	
C44	0.68267 (16)	0.09932 (13)	0.34798 (7)	0.0157 (4)	
C45	0.60343 (17)	0.06770 (13)	0.38805 (8)	0.0190 (4)	
H45A	0.6322	0.0836	0.4208	0.023*	
H45B	0.5340	0.0978	0.3837	0.023*	
C46	0.58308 (17)	−0.03024 (14)	0.38704 (8)	0.0203 (4)	
H46A	0.5362	−0.0443	0.3587	0.024*	
H46B	0.5448	−0.0472	0.4175	0.024*	
C47	0.68880 (17)	−0.08248 (13)	0.38292 (8)	0.0187 (4)	
C48	0.74607 (17)	−0.05958 (13)	0.33453 (7)	0.0177 (4)	
H48	0.8143	−0.0934	0.3345	0.021*	
C49	0.68355 (18)	−0.08948 (13)	0.28927 (8)	0.0208 (4)	
H49A	0.7240	−0.0745	0.2591	0.025*	
H49B	0.6132	−0.0599	0.2880	0.025*	
C50	0.66565 (19)	−0.18817 (14)	0.29135 (9)	0.0244 (5)	
C51	0.6154 (2)	−0.21427 (14)	0.34126 (9)	0.0270 (5)	
H51A	0.5400	−0.1940	0.3420	0.032*	
H51B	0.6139	−0.2777	0.3432	0.032*	
C52	0.67319 (19)	−0.17968 (14)	0.38724 (9)	0.0247 (5)	
H52A	0.6302	−0.1928	0.4169	0.030*	
H52B	0.7440	−0.2081	0.3907	0.030*	
C53	0.70908 (18)	0.50268 (14)	0.26005 (8)	0.0241 (5)	
H53A	0.7244	0.5637	0.2549	0.036*	
H53B	0.6346	0.4958	0.2710	0.036*	
H53C	0.7196	0.4714	0.2291	0.036*	
C54	0.77561 (19)	0.52216 (14)	0.34640 (8)	0.0238 (5)	
H54A	0.8234	0.4998	0.3721	0.036*	
H54B	0.7012	0.5207	0.3581	0.036*	
H54C	0.7957	0.5815	0.3386	0.036*	
C55	0.92374 (19)	0.34467 (15)	0.36309 (8)	0.0234 (5)	
H55A	0.9649	0.3957	0.3536	0.035*	
H55B	0.9732	0.2996	0.3741	0.035*	
H55C	0.8744	0.3593	0.3899	0.035*	
C56	0.7498 (2)	0.21010 (14)	0.41173 (8)	0.0227 (5)	
H56A	0.7843	0.2661	0.4160	0.034*	
H56B	0.7988	0.1647	0.4227	0.034*	
H56C	0.6837	0.2080	0.4313	0.034*	
C57	0.62456 (18)	0.09857 (13)	0.29812 (8)	0.0187 (4)	
H57	0.6663	0.1059	0.2692	0.022*	
C58	0.76600 (18)	−0.04329 (13)	0.41932 (8)	0.0208 (4)	
C59	0.5866 (2)	−0.21247 (16)	0.25044 (10)	0.0358 (6)	
H59A	0.6160	−0.1951	0.2185	0.054*	
H59B	0.5179	−0.1832	0.2560	0.054*	
H59C	0.5751	−0.2747	0.2507	0.054*	
C60	0.7720 (2)	−0.23523 (15)	0.28285 (10)	0.0312 (6)	
H60A	0.7589	−0.2972	0.2816	0.047*	
H60B	0.8216	−0.2223	0.3099	0.047*	
H60C	0.8036	−0.2163	0.2516	0.047*	

### Atomic displacement parameters (Å^2^)

**Table:**

	*U*^11^	*U*^22^	*U*^33^	*U*^12^	*U*^13^	*U*^23^
O1	0.0551 (13)	0.0287 (10)	0.0571 (13)	0.0092 (9)	0.0241 (11)	−0.0036 (9)
O2	0.0134 (7)	0.0236 (8)	0.0234 (8)	0.0006 (6)	−0.0006 (6)	0.0030 (6)
O3	0.0178 (7)	0.0174 (7)	0.0208 (8)	0.0013 (6)	0.0064 (6)	0.0019 (6)
O4	0.0199 (8)	0.0227 (8)	0.0428 (10)	0.0033 (6)	0.0124 (8)	0.0032 (7)
O5	0.0394 (10)	0.0310 (9)	0.0175 (8)	−0.0095 (8)	−0.0004 (7)	−0.0018 (7)
O6	0.0283 (9)	0.0209 (8)	0.0298 (9)	−0.0069 (7)	0.0069 (7)	−0.0031 (7)
O7	0.0213 (8)	0.0233 (8)	0.0166 (7)	0.0007 (6)	0.0041 (6)	−0.0003 (6)
O8	0.0218 (7)	0.0194 (7)	0.0151 (7)	−0.0010 (6)	−0.0020 (6)	0.0032 (6)
O9	0.0332 (9)	0.0328 (9)	0.0201 (8)	−0.0006 (8)	−0.0014 (7)	0.0096 (7)
O10	0.0188 (8)	0.0247 (8)	0.0338 (9)	−0.0008 (6)	−0.0039 (7)	0.0014 (7)
C1	0.0212 (10)	0.0185 (10)	0.0278 (12)	−0.0006 (9)	0.0013 (9)	0.0035 (9)
C2	0.0281 (12)	0.0206 (11)	0.0340 (13)	0.0022 (9)	0.0061 (10)	0.0041 (10)
C3	0.0382 (14)	0.0152 (11)	0.0369 (14)	−0.0014 (10)	0.0175 (11)	0.0020 (10)
C4	0.0447 (15)	0.0177 (11)	0.0227 (12)	−0.0048 (10)	0.0050 (11)	−0.0006 (9)
C5	0.0279 (11)	0.0190 (10)	0.0180 (10)	−0.0045 (9)	0.0040 (9)	−0.0010 (8)
C6	0.0328 (12)	0.0224 (11)	0.0216 (11)	−0.0105 (10)	−0.0024 (10)	−0.0024 (9)
C7	0.0275 (12)	0.0235 (11)	0.0224 (11)	−0.0094 (9)	−0.0078 (9)	0.0007 (9)
C8	0.0170 (10)	0.0178 (10)	0.0189 (10)	−0.0023 (8)	−0.0024 (8)	0.0000 (8)
C9	0.0171 (10)	0.0166 (10)	0.0149 (10)	−0.0028 (8)	0.0006 (8)	−0.0014 (8)
C10	0.0182 (10)	0.0163 (10)	0.0187 (10)	−0.0027 (8)	0.0040 (8)	−0.0004 (8)
C11	0.0160 (9)	0.0183 (10)	0.0166 (10)	0.0008 (8)	−0.0022 (8)	−0.0015 (8)
C12	0.0173 (10)	0.0159 (10)	0.0164 (10)	0.0007 (8)	−0.0005 (8)	−0.0017 (8)
C13	0.0141 (9)	0.0177 (10)	0.0152 (10)	0.0001 (8)	0.0027 (8)	−0.0004 (8)
C14	0.0149 (10)	0.0200 (10)	0.0169 (10)	−0.0031 (8)	−0.0020 (8)	0.0003 (8)
C15	0.0190 (11)	0.0259 (11)	0.0266 (12)	−0.0044 (9)	−0.0063 (9)	0.0054 (9)
C16	0.0179 (11)	0.0258 (11)	0.0308 (13)	−0.0017 (9)	−0.0055 (9)	0.0096 (10)
C17	0.0161 (10)	0.0204 (10)	0.0239 (11)	0.0011 (8)	0.0018 (9)	0.0031 (9)
C18	0.0158 (10)	0.0161 (9)	0.0178 (10)	−0.0011 (8)	0.0002 (8)	−0.0003 (8)
C19	0.0175 (10)	0.0212 (10)	0.0150 (10)	−0.0034 (8)	−0.0002 (8)	0.0003 (8)
C20	0.0199 (10)	0.0218 (11)	0.0196 (10)	−0.0050 (9)	−0.0001 (9)	0.0014 (9)
C21	0.0257 (11)	0.0206 (10)	0.0292 (12)	−0.0042 (9)	−0.0026 (10)	0.0069 (9)
C22	0.0223 (11)	0.0190 (11)	0.0375 (14)	0.0016 (9)	0.0031 (10)	0.0068 (10)
C23	0.082 (2)	0.0208 (12)	0.0325 (14)	−0.0022 (14)	0.0119 (15)	−0.0060 (11)
C24	0.0432 (15)	0.0216 (12)	0.0339 (14)	−0.0113 (11)	0.0007 (12)	0.0006 (10)
C25	0.0232 (11)	0.0217 (11)	0.0184 (10)	−0.0021 (9)	0.0018 (9)	0.0010 (9)
C26	0.0161 (10)	0.0233 (11)	0.0312 (13)	−0.0022 (9)	−0.0005 (9)	0.0054 (10)
C27	0.0256 (11)	0.0141 (9)	0.0207 (11)	−0.0046 (9)	−0.0001 (9)	−0.0027 (8)
C28	0.0180 (10)	0.0162 (10)	0.0289 (12)	−0.0006 (8)	0.0015 (9)	0.0011 (9)
C29	0.0289 (12)	0.0299 (12)	0.0256 (12)	−0.0106 (10)	0.0020 (10)	0.0032 (10)
C30	0.0281 (12)	0.0236 (11)	0.0251 (12)	−0.0086 (9)	−0.0026 (10)	−0.0007 (9)
C31	0.0197 (10)	0.0184 (10)	0.0241 (11)	0.0005 (8)	0.0059 (9)	0.0008 (9)
C32	0.0256 (11)	0.0201 (10)	0.0195 (11)	−0.0014 (9)	0.0057 (9)	0.0007 (9)
C33	0.0231 (11)	0.0181 (10)	0.0169 (10)	−0.0001 (9)	0.0011 (8)	0.0038 (8)
C34	0.0232 (10)	0.0131 (10)	0.0203 (10)	−0.0007 (8)	0.0044 (9)	0.0007 (8)
C35	0.0173 (10)	0.0140 (9)	0.0183 (10)	0.0011 (8)	0.0020 (8)	0.0009 (8)
C36	0.0208 (10)	0.0150 (10)	0.0270 (12)	0.0020 (8)	0.0051 (9)	−0.0017 (9)
C37	0.0164 (10)	0.0155 (10)	0.0268 (12)	0.0014 (8)	0.0068 (9)	−0.0001 (9)
C38	0.0174 (10)	0.0148 (9)	0.0170 (10)	−0.0011 (8)	0.0018 (8)	−0.0017 (8)
C39	0.0136 (9)	0.0166 (10)	0.0159 (10)	−0.0005 (8)	0.0009 (8)	−0.0007 (8)
C40	0.0163 (10)	0.0158 (10)	0.0171 (10)	−0.0004 (8)	−0.0001 (8)	−0.0003 (8)
C41	0.0149 (10)	0.0184 (10)	0.0203 (11)	0.0002 (8)	0.0008 (8)	0.0015 (8)
C42	0.0150 (9)	0.0186 (10)	0.0173 (10)	0.0019 (8)	−0.0005 (8)	0.0009 (8)
C43	0.0158 (9)	0.0176 (10)	0.0143 (9)	0.0009 (8)	−0.0011 (8)	0.0019 (8)
C44	0.0174 (9)	0.0149 (9)	0.0148 (10)	−0.0002 (8)	0.0031 (8)	−0.0003 (8)
C45	0.0192 (10)	0.0169 (10)	0.0209 (11)	−0.0002 (8)	0.0041 (8)	0.0019 (8)
C46	0.0202 (10)	0.0188 (10)	0.0218 (11)	−0.0005 (9)	0.0043 (9)	0.0016 (9)
C47	0.0211 (10)	0.0168 (10)	0.0183 (10)	0.0005 (8)	0.0027 (9)	0.0019 (8)
C48	0.0189 (10)	0.0153 (9)	0.0189 (10)	0.0014 (8)	0.0022 (8)	0.0009 (8)
C49	0.0248 (11)	0.0164 (10)	0.0213 (11)	−0.0020 (9)	0.0007 (9)	−0.0032 (9)
C50	0.0275 (12)	0.0169 (10)	0.0288 (12)	−0.0033 (9)	0.0020 (10)	−0.0019 (9)
C51	0.0306 (12)	0.0175 (10)	0.0331 (13)	−0.0048 (9)	0.0074 (11)	0.0016 (10)
C52	0.0253 (11)	0.0182 (11)	0.0306 (13)	−0.0013 (9)	0.0059 (10)	0.0033 (9)
C53	0.0267 (11)	0.0181 (10)	0.0276 (12)	0.0015 (9)	−0.0011 (10)	0.0027 (9)
C54	0.0278 (11)	0.0182 (10)	0.0254 (11)	−0.0023 (9)	0.0053 (10)	−0.0030 (9)
C55	0.0253 (11)	0.0211 (11)	0.0238 (12)	−0.0037 (9)	−0.0058 (9)	0.0018 (9)
C56	0.0317 (12)	0.0186 (10)	0.0178 (10)	−0.0038 (9)	0.0025 (9)	−0.0007 (8)
C57	0.0217 (11)	0.0138 (10)	0.0207 (11)	−0.0001 (8)	0.0003 (9)	−0.0008 (8)
C58	0.0220 (11)	0.0207 (11)	0.0196 (11)	0.0039 (9)	0.0023 (9)	0.0033 (9)
C59	0.0450 (15)	0.0247 (12)	0.0376 (14)	−0.0110 (11)	0.0002 (13)	−0.0069 (11)
C60	0.0365 (14)	0.0181 (11)	0.0391 (14)	0.0009 (10)	0.0091 (12)	−0.0041 (10)

### Geometric parameters (Å, º)

**Table:**

O1—C3	1.218 (3)	C27—H27	0.9500
O2—C12	1.429 (2)	C29—H29A	0.9800
O2—H2	0.86 (3)	C29—H29B	0.9800
O3—C28	1.356 (3)	C29—H29C	0.9800
O3—C13	1.486 (2)	C30—H30A	0.9800
O4—C28	1.213 (3)	C30—H30B	0.9800
O5—C27	1.210 (3)	C30—H30C	0.9800
O6—C33	1.223 (3)	C31—C32	1.545 (3)
O7—C42	1.438 (2)	C31—C40	1.548 (3)
O7—H7	0.90 (3)	C31—H31A	0.9900
O8—C58	1.375 (3)	C31—H31B	0.9900
O8—C43	1.484 (2)	C32—C33	1.504 (3)
O9—C58	1.204 (3)	C32—H32A	0.9900
O10—C57	1.214 (3)	C32—H32B	0.9900
C1—C2	1.539 (3)	C33—C34	1.526 (3)
C1—C10	1.549 (3)	C34—C54	1.542 (3)
C1—H1A	0.9900	C34—C53	1.550 (3)
C1—H1B	0.9900	C34—C35	1.570 (3)
C2—C3	1.507 (4)	C35—C36	1.532 (3)
C2—H2A	0.9900	C35—C40	1.547 (3)
C2—H2B	0.9900	C35—H35	1.0000
C3—C4	1.534 (4)	C36—C37	1.534 (3)
C4—C24	1.544 (3)	C36—H36A	0.9900
C4—C23	1.544 (3)	C36—H36B	0.9900
C4—C5	1.567 (3)	C37—C38	1.544 (3)
C5—C6	1.531 (3)	C37—H37A	0.9900
C5—C10	1.553 (3)	C37—H37B	0.9900
C5—H5	1.0000	C38—C56	1.539 (3)
C6—C7	1.532 (3)	C38—C39	1.565 (3)
C6—H6A	0.9900	C38—C44	1.613 (3)
C6—H6B	0.9900	C39—C41	1.533 (3)
C7—C8	1.544 (3)	C39—C40	1.573 (3)
C7—H7A	0.9900	C39—H39	1.0000
C7—H7B	0.9900	C40—C55	1.551 (3)
C8—C26	1.547 (3)	C41—C42	1.517 (3)
C8—C9	1.571 (3)	C41—H41A	0.9900
C8—C14	1.614 (3)	C41—H41B	0.9900
C9—C11	1.538 (3)	C42—C43	1.519 (3)
C9—C10	1.574 (3)	C42—H42	1.0000
C9—H9	1.0000	C43—C48	1.546 (3)
C10—C25	1.544 (3)	C43—C44	1.574 (3)
C11—C12	1.529 (3)	C44—C57	1.536 (3)
C11—H11A	0.9900	C44—C45	1.550 (3)
C11—H11B	0.9900	C45—C46	1.547 (3)
C12—C13	1.525 (3)	C45—H45A	0.9900
C12—H12	1.0000	C45—H45B	0.9900
C13—C18	1.550 (3)	C46—C47	1.551 (3)
C13—C14	1.568 (3)	C46—H46A	0.9900
C14—C27	1.541 (3)	C46—H46B	0.9900
C14—C15	1.553 (3)	C47—C58	1.508 (3)
C15—C16	1.541 (3)	C47—C52	1.531 (3)
C15—H15A	0.9900	C47—C48	1.538 (3)
C15—H15B	0.9900	C48—C49	1.528 (3)
C16—C17	1.550 (3)	C48—H48	1.0000
C16—H16A	0.9900	C49—C50	1.555 (3)
C16—H16B	0.9900	C49—H49A	0.9900
C17—C28	1.502 (3)	C49—H49B	0.9900
C17—C22	1.528 (3)	C50—C60	1.530 (3)
C17—C18	1.534 (3)	C50—C59	1.532 (3)
C18—C19	1.526 (3)	C50—C51	1.548 (3)
C18—H18	1.0000	C51—C52	1.539 (3)
C19—C20	1.555 (3)	C51—H51A	0.9900
C19—H19A	0.9900	C51—H51B	0.9900
C19—H19B	0.9900	C52—H52A	0.9900
C20—C29	1.529 (3)	C52—H52B	0.9900
C20—C30	1.531 (3)	C53—H53A	0.9800
C20—C21	1.547 (3)	C53—H53B	0.9800
C21—C22	1.536 (3)	C53—H53C	0.9800
C21—H21A	0.9900	C54—H54A	0.9800
C21—H21B	0.9900	C54—H54B	0.9800
C22—H22A	0.9900	C54—H54C	0.9800
C22—H22B	0.9900	C55—H55A	0.9800
C23—H23A	0.9800	C55—H55B	0.9800
C23—H23B	0.9800	C55—H55C	0.9800
C23—H23C	0.9800	C56—H56A	0.9800
C24—H24A	0.9800	C56—H56B	0.9800
C24—H24B	0.9800	C56—H56C	0.9800
C24—H24C	0.9800	C57—H57	0.9500
C25—H25A	0.9800	C59—H59A	0.9800
C25—H25B	0.9800	C59—H59B	0.9800
C25—H25C	0.9800	C59—H59C	0.9800
C26—H26A	0.9800	C60—H60A	0.9800
C26—H26B	0.9800	C60—H60B	0.9800
C26—H26C	0.9800	C60—H60C	0.9800
			
C12—O2—H2	107.6 (18)	H30A—C30—H30C	109.5
C28—O3—C13	109.56 (15)	H30B—C30—H30C	109.5
C42—O7—H7	106.5 (17)	C32—C31—C40	113.90 (17)
C58—O8—C43	109.85 (15)	C32—C31—H31A	108.8
C2—C1—C10	113.53 (18)	C40—C31—H31A	108.8
C2—C1—H1A	108.9	C32—C31—H31B	108.8
C10—C1—H1A	108.9	C40—C31—H31B	108.8
C2—C1—H1B	108.9	H31A—C31—H31B	107.7
C10—C1—H1B	108.9	C33—C32—C31	114.20 (17)
H1A—C1—H1B	107.7	C33—C32—H32A	108.7
C3—C2—C1	113.6 (2)	C31—C32—H32A	108.7
C3—C2—H2A	108.8	C33—C32—H32B	108.7
C1—C2—H2A	108.8	C31—C32—H32B	108.7
C3—C2—H2B	108.8	H32A—C32—H32B	107.6
C1—C2—H2B	108.8	O6—C33—C32	122.40 (19)
H2A—C2—H2B	107.7	O6—C33—C34	121.72 (19)
O1—C3—C2	120.4 (3)	C32—C33—C34	115.72 (18)
O1—C3—C4	121.8 (2)	C33—C34—C54	109.62 (18)
C2—C3—C4	117.7 (2)	C33—C34—C53	104.90 (17)
C3—C4—C24	107.71 (19)	C54—C34—C53	108.28 (17)
C3—C4—C23	108.6 (2)	C33—C34—C35	110.20 (17)
C24—C4—C23	107.6 (2)	C54—C34—C35	115.13 (17)
C3—C4—C5	109.11 (19)	C53—C34—C35	108.18 (17)
C24—C4—C5	114.3 (2)	C36—C35—C40	112.09 (16)
C23—C4—C5	109.41 (19)	C36—C35—C34	112.83 (16)
C6—C5—C10	110.54 (18)	C40—C35—C34	114.22 (17)
C6—C5—C4	113.37 (19)	C36—C35—H35	105.6
C10—C5—C4	118.14 (18)	C40—C35—H35	105.6
C6—C5—H5	104.4	C34—C35—H35	105.6
C10—C5—H5	104.4	C35—C36—C37	111.09 (17)
C4—C5—H5	104.4	C35—C36—H36A	109.4
C5—C6—C7	109.83 (18)	C37—C36—H36A	109.4
C5—C6—H6A	109.7	C35—C36—H36B	109.4
C7—C6—H6A	109.7	C37—C36—H36B	109.4
C5—C6—H6B	109.7	H36A—C36—H36B	108.0
C7—C6—H6B	109.7	C36—C37—C38	112.67 (17)
H6A—C6—H6B	108.2	C36—C37—H37A	109.1
C6—C7—C8	113.35 (18)	C38—C37—H37A	109.1
C6—C7—H7A	108.9	C36—C37—H37B	109.1
C8—C7—H7A	108.9	C38—C37—H37B	109.1
C6—C7—H7B	108.9	H37A—C37—H37B	107.8
C8—C7—H7B	108.9	C56—C38—C37	108.07 (17)
H7A—C7—H7B	107.7	C56—C38—C39	112.67 (17)
C7—C8—C26	107.35 (17)	C37—C38—C39	107.11 (16)
C7—C8—C9	108.87 (17)	C56—C38—C44	110.11 (16)
C26—C8—C9	112.54 (17)	C37—C38—C44	109.66 (16)
C7—C8—C14	108.87 (16)	C39—C38—C44	109.14 (16)
C26—C8—C14	111.02 (17)	C41—C39—C38	110.99 (16)
C9—C8—C14	108.12 (16)	C41—C39—C40	112.64 (16)
C11—C9—C8	109.04 (16)	C38—C39—C40	117.86 (16)
C11—C9—C10	114.63 (16)	C41—C39—H39	104.6
C8—C9—C10	117.24 (17)	C38—C39—H39	104.6
C11—C9—H9	104.9	C40—C39—H39	104.6
C8—C9—H9	104.9	C35—C40—C31	108.78 (16)
C10—C9—H9	104.9	C35—C40—C55	112.50 (17)
C25—C10—C1	107.56 (17)	C31—C40—C55	106.38 (17)
C25—C10—C5	114.81 (17)	C35—C40—C39	107.96 (16)
C1—C10—C5	107.07 (18)	C31—C40—C39	106.22 (16)
C25—C10—C9	113.22 (17)	C55—C40—C39	114.67 (17)
C1—C10—C9	107.26 (16)	C42—C41—C39	109.93 (17)
C5—C10—C9	106.52 (16)	C42—C41—H41A	109.7
C12—C11—C9	110.20 (16)	C39—C41—H41A	109.7
C12—C11—H11A	109.6	C42—C41—H41B	109.7
C9—C11—H11A	109.6	C39—C41—H41B	109.7
C12—C11—H11B	109.6	H41A—C41—H41B	108.2
C9—C11—H11B	109.6	O7—C42—C41	111.71 (17)
H11A—C11—H11B	108.1	O7—C42—C43	107.19 (16)
O2—C12—C13	106.06 (16)	C41—C42—C43	112.14 (17)
O2—C12—C11	111.91 (16)	O7—C42—H42	108.6
C13—C12—C11	112.64 (17)	C41—C42—H42	108.6
O2—C12—H12	108.7	C43—C42—H42	108.6
C13—C12—H12	108.7	O8—C43—C42	103.83 (15)
C11—C12—H12	108.7	O8—C43—C48	99.81 (15)
O3—C13—C12	104.34 (15)	C42—C43—C48	113.37 (17)
O3—C13—C18	99.85 (15)	O8—C43—C44	106.62 (15)
C12—C13—C18	113.44 (17)	C42—C43—C44	116.33 (16)
O3—C13—C14	106.49 (15)	C48—C43—C44	114.52 (16)
C12—C13—C14	115.98 (17)	C57—C44—C45	108.53 (17)
C18—C13—C14	114.50 (16)	C57—C44—C43	108.65 (16)
C27—C14—C15	108.79 (18)	C45—C44—C43	107.88 (16)
C27—C14—C13	107.55 (16)	C57—C44—C38	106.63 (16)
C15—C14—C13	108.05 (17)	C45—C44—C38	112.72 (16)
C27—C14—C8	107.53 (16)	C43—C44—C38	112.32 (16)
C15—C14—C8	112.05 (16)	C46—C45—C44	113.91 (17)
C13—C14—C8	112.71 (16)	C46—C45—H45A	108.8
C16—C15—C14	113.45 (18)	C44—C45—H45A	108.8
C16—C15—H15A	108.9	C46—C45—H45B	108.8
C14—C15—H15A	108.9	C44—C45—H45B	108.8
C16—C15—H15B	108.9	H45A—C45—H45B	107.7
C14—C15—H15B	108.9	C45—C46—C47	112.33 (17)
H15A—C15—H15B	107.7	C45—C46—H46A	109.1
C15—C16—C17	113.14 (18)	C47—C46—H46A	109.1
C15—C16—H16A	109.0	C45—C46—H46B	109.1
C17—C16—H16A	109.0	C47—C46—H46B	109.1
C15—C16—H16B	109.0	H46A—C46—H46B	107.9
C17—C16—H16B	109.0	C58—C47—C52	115.53 (18)
H16A—C16—H16B	107.8	C58—C47—C48	99.88 (17)
C28—C17—C22	114.91 (19)	C52—C47—C48	110.73 (17)
C28—C17—C18	98.85 (17)	C58—C47—C46	106.30 (17)
C22—C17—C18	111.47 (18)	C52—C47—C46	113.94 (18)
C28—C17—C16	107.05 (17)	C48—C47—C46	109.46 (17)
C22—C17—C16	113.57 (19)	C49—C48—C47	112.42 (17)
C18—C17—C16	109.96 (18)	C49—C48—C43	125.55 (17)
C19—C18—C17	112.18 (17)	C47—C48—C43	100.01 (16)
C19—C18—C13	126.18 (17)	C49—C48—H48	105.8
C17—C18—C13	99.71 (16)	C47—C48—H48	105.8
C19—C18—H18	105.7	C43—C48—H48	105.8
C17—C18—H18	105.7	C48—C49—C50	110.19 (18)
C13—C18—H18	105.7	C48—C49—H49A	109.6
C18—C19—C20	110.06 (17)	C50—C49—H49A	109.6
C18—C19—H19A	109.6	C48—C49—H49B	109.6
C20—C19—H19A	109.6	C50—C49—H49B	109.6
C18—C19—H19B	109.6	H49A—C49—H49B	108.1
C20—C19—H19B	109.6	C60—C50—C59	109.1 (2)
H19A—C19—H19B	108.2	C60—C50—C51	110.8 (2)
C29—C20—C30	108.42 (18)	C59—C50—C51	108.13 (19)
C29—C20—C21	108.53 (18)	C60—C50—C49	110.14 (19)
C30—C20—C21	110.72 (19)	C59—C50—C49	108.05 (19)
C29—C20—C19	107.54 (18)	C51—C50—C49	110.47 (18)
C30—C20—C19	110.98 (18)	C52—C51—C50	115.50 (18)
C21—C20—C19	110.54 (17)	C52—C51—H51A	108.4
C22—C21—C20	114.17 (18)	C50—C51—H51A	108.4
C22—C21—H21A	108.7	C52—C51—H51B	108.4
C20—C21—H21A	108.7	C50—C51—H51B	108.4
C22—C21—H21B	108.7	H51A—C51—H51B	107.5
C20—C21—H21B	108.7	C47—C52—C51	110.08 (19)
H21A—C21—H21B	107.6	C47—C52—H52A	109.6
C17—C22—C21	109.58 (19)	C51—C52—H52A	109.6
C17—C22—H22A	109.8	C47—C52—H52B	109.6
C21—C22—H22A	109.8	C51—C52—H52B	109.6
C17—C22—H22B	109.8	H52A—C52—H52B	108.2
C21—C22—H22B	109.8	C34—C53—H53A	109.5
H22A—C22—H22B	108.2	C34—C53—H53B	109.5
C4—C23—H23A	109.5	H53A—C53—H53B	109.5
C4—C23—H23B	109.5	C34—C53—H53C	109.5
H23A—C23—H23B	109.5	H53A—C53—H53C	109.5
C4—C23—H23C	109.5	H53B—C53—H53C	109.5
H23A—C23—H23C	109.5	C34—C54—H54A	109.5
H23B—C23—H23C	109.5	C34—C54—H54B	109.5
C4—C24—H24A	109.5	H54A—C54—H54B	109.5
C4—C24—H24B	109.5	C34—C54—H54C	109.5
H24A—C24—H24B	109.5	H54A—C54—H54C	109.5
C4—C24—H24C	109.5	H54B—C54—H54C	109.5
H24A—C24—H24C	109.5	C40—C55—H55A	109.5
H24B—C24—H24C	109.5	C40—C55—H55B	109.5
C10—C25—H25A	109.5	H55A—C55—H55B	109.5
C10—C25—H25B	109.5	C40—C55—H55C	109.5
H25A—C25—H25B	109.5	H55A—C55—H55C	109.5
C10—C25—H25C	109.5	H55B—C55—H55C	109.5
H25A—C25—H25C	109.5	C38—C56—H56A	109.5
H25B—C25—H25C	109.5	C38—C56—H56B	109.5
C8—C26—H26A	109.5	H56A—C56—H56B	109.5
C8—C26—H26B	109.5	C38—C56—H56C	109.5
H26A—C26—H26B	109.5	H56A—C56—H56C	109.5
C8—C26—H26C	109.5	H56B—C56—H56C	109.5
H26A—C26—H26C	109.5	O10—C57—C44	123.6 (2)
H26B—C26—H26C	109.5	O10—C57—H57	118.2
O5—C27—C14	123.7 (2)	C44—C57—H57	118.2
O5—C27—H27	118.2	O9—C58—O8	120.8 (2)
C14—C27—H27	118.2	O9—C58—C47	130.4 (2)
O4—C28—O3	120.6 (2)	O8—C58—C47	108.69 (17)
O4—C28—C17	129.8 (2)	C50—C59—H59A	109.5
O3—C28—C17	109.52 (18)	C50—C59—H59B	109.5
C20—C29—H29A	109.5	H59A—C59—H59B	109.5
C20—C29—H29B	109.5	C50—C59—H59C	109.5
H29A—C29—H29B	109.5	H59A—C59—H59C	109.5
C20—C29—H29C	109.5	H59B—C59—H59C	109.5
H29A—C29—H29C	109.5	C50—C60—H60A	109.5
H29B—C29—H29C	109.5	C50—C60—H60B	109.5
C20—C30—H30A	109.5	H60A—C60—H60B	109.5
C20—C30—H30B	109.5	C50—C60—H60C	109.5
H30A—C30—H30B	109.5	H60A—C60—H60C	109.5
C20—C30—H30C	109.5	H60B—C60—H60C	109.5
			
C10—C1—C2—C3	−52.1 (3)	C40—C31—C32—C33	20.7 (3)
C1—C2—C3—O1	−136.9 (2)	C31—C32—C33—O6	129.2 (2)
C1—C2—C3—C4	46.1 (3)	C31—C32—C33—C34	−55.3 (3)
O1—C3—C4—C24	−94.2 (3)	O6—C33—C34—C54	−29.2 (3)
C2—C3—C4—C24	82.8 (2)	C32—C33—C34—C54	155.27 (18)
O1—C3—C4—C23	22.1 (3)	O6—C33—C34—C53	86.9 (2)
C2—C3—C4—C23	−161.0 (2)	C32—C33—C34—C53	−88.7 (2)
O1—C3—C4—C5	141.2 (2)	O6—C33—C34—C35	−156.9 (2)
C2—C3—C4—C5	−41.8 (3)	C32—C33—C34—C35	27.6 (2)
C3—C4—C5—C6	179.02 (18)	C33—C34—C35—C36	160.31 (17)
C24—C4—C5—C6	58.4 (3)	C54—C34—C35—C36	35.7 (3)
C23—C4—C5—C6	−62.3 (3)	C53—C34—C35—C36	−85.5 (2)
C3—C4—C5—C10	47.4 (3)	C33—C34—C35—C40	30.8 (2)
C24—C4—C5—C10	−73.3 (3)	C54—C34—C35—C40	−93.8 (2)
C23—C4—C5—C10	166.0 (2)	C53—C34—C35—C40	144.92 (18)
C10—C5—C6—C7	−64.5 (2)	C40—C35—C36—C37	−59.4 (2)
C4—C5—C6—C7	160.22 (19)	C34—C35—C36—C37	169.95 (18)
C5—C6—C7—C8	59.5 (3)	C35—C36—C37—C38	60.4 (2)
C6—C7—C8—C26	72.8 (2)	C36—C37—C38—C56	68.0 (2)
C6—C7—C8—C9	−49.3 (2)	C36—C37—C38—C39	−53.6 (2)
C6—C7—C8—C14	−166.96 (18)	C36—C37—C38—C44	−171.93 (17)
C7—C8—C9—C11	−179.94 (17)	C56—C38—C39—C41	64.3 (2)
C26—C8—C9—C11	61.2 (2)	C37—C38—C39—C41	−176.96 (16)
C14—C8—C9—C11	−61.8 (2)	C44—C38—C39—C41	−58.3 (2)
C7—C8—C9—C10	47.7 (2)	C56—C38—C39—C40	−67.7 (2)
C26—C8—C9—C10	−71.2 (2)	C37—C38—C39—C40	51.0 (2)
C14—C8—C9—C10	165.84 (16)	C44—C38—C39—C40	169.70 (16)
C2—C1—C10—C25	−70.1 (2)	C36—C35—C40—C31	167.31 (17)
C2—C1—C10—C5	53.8 (2)	C34—C35—C40—C31	−62.8 (2)
C2—C1—C10—C9	167.77 (18)	C36—C35—C40—C55	−75.1 (2)
C6—C5—C10—C25	−67.3 (2)	C34—C35—C40—C55	54.8 (2)
C4—C5—C10—C25	65.6 (3)	C36—C35—C40—C39	52.4 (2)
C6—C5—C10—C1	173.35 (17)	C34—C35—C40—C39	−177.64 (16)
C4—C5—C10—C1	−53.7 (2)	C32—C31—C40—C35	34.5 (2)
C6—C5—C10—C9	58.8 (2)	C32—C31—C40—C55	−86.9 (2)
C4—C5—C10—C9	−168.26 (19)	C32—C31—C40—C39	150.53 (18)
C11—C9—C10—C25	−55.1 (2)	C41—C39—C40—C35	177.88 (17)
C8—C9—C10—C25	74.7 (2)	C38—C39—C40—C35	−50.9 (2)
C11—C9—C10—C1	63.4 (2)	C41—C39—C40—C31	61.3 (2)
C8—C9—C10—C1	−166.82 (17)	C38—C39—C40—C31	−167.41 (17)
C11—C9—C10—C5	177.79 (17)	C41—C39—C40—C55	−55.8 (2)
C8—C9—C10—C5	−52.4 (2)	C38—C39—C40—C55	75.4 (2)
C8—C9—C11—C12	66.6 (2)	C38—C39—C41—C42	65.1 (2)
C10—C9—C11—C12	−159.71 (17)	C40—C39—C41—C42	−160.27 (17)
C9—C11—C12—O2	62.9 (2)	C39—C41—C42—O7	62.6 (2)
C9—C11—C12—C13	−56.5 (2)	C39—C41—C42—C43	−57.8 (2)
C28—O3—C13—C12	−143.36 (17)	C58—O8—C43—C42	−145.75 (16)
C28—O3—C13—C18	−25.91 (19)	C58—O8—C43—C48	−28.54 (19)
C28—O3—C13—C14	93.46 (18)	C58—O8—C43—C44	90.88 (18)
O2—C12—C13—O3	165.17 (14)	O7—C42—C43—O8	167.71 (15)
C11—C12—C13—O3	−72.09 (19)	C41—C42—C43—O8	−69.34 (19)
O2—C12—C13—C18	57.5 (2)	O7—C42—C43—C48	60.4 (2)
C11—C12—C13—C18	−179.75 (16)	C41—C42—C43—C48	−176.65 (16)
O2—C12—C13—C14	−78.1 (2)	O7—C42—C43—C44	−75.5 (2)
C11—C12—C13—C14	44.7 (2)	C41—C42—C43—C44	47.4 (2)
O3—C13—C14—C27	−167.75 (15)	O8—C43—C44—C57	−168.79 (15)
C12—C13—C14—C27	76.7 (2)	C42—C43—C44—C57	76.0 (2)
C18—C13—C14—C27	−58.4 (2)	C48—C43—C44—C57	−59.4 (2)
O3—C13—C14—C15	−50.5 (2)	O8—C43—C44—C45	−51.3 (2)
C12—C13—C14—C15	−166.03 (17)	C42—C43—C44—C45	−166.53 (17)
C18—C13—C14—C15	58.9 (2)	C48—C43—C44—C45	58.0 (2)
O3—C13—C14—C8	73.90 (19)	O8—C43—C44—C38	73.51 (19)
C12—C13—C14—C8	−41.7 (2)	C42—C43—C44—C38	−41.7 (2)
C18—C13—C14—C8	−176.77 (16)	C48—C43—C44—C38	−177.12 (16)
C7—C8—C14—C27	48.9 (2)	C56—C38—C44—C57	162.64 (17)
C26—C8—C14—C27	166.90 (17)	C37—C38—C44—C57	43.8 (2)
C9—C8—C14—C27	−69.2 (2)	C39—C38—C44—C57	−73.20 (19)
C7—C8—C14—C15	−70.6 (2)	C56—C38—C44—C45	43.7 (2)
C26—C8—C14—C15	47.4 (2)	C37—C38—C44—C45	−75.1 (2)
C9—C8—C14—C15	171.30 (17)	C39—C38—C44—C45	167.81 (17)
C7—C8—C14—C13	167.30 (17)	C56—C38—C44—C43	−78.5 (2)
C26—C8—C14—C13	−74.7 (2)	C37—C38—C44—C43	162.75 (16)
C9—C8—C14—C13	49.2 (2)	C39—C38—C44—C43	45.7 (2)
C27—C14—C15—C16	73.7 (2)	C57—C44—C45—C46	74.7 (2)
C13—C14—C15—C16	−42.8 (2)	C43—C44—C45—C46	−42.9 (2)
C8—C14—C15—C16	−167.52 (18)	C38—C44—C45—C46	−167.45 (17)
C14—C15—C16—C17	45.9 (3)	C44—C45—C46—C47	46.9 (2)
C15—C16—C17—C28	45.8 (2)	C45—C46—C47—C58	45.5 (2)
C15—C16—C17—C22	173.74 (19)	C45—C46—C47—C52	173.92 (19)
C15—C16—C17—C18	−60.6 (2)	C45—C46—C47—C48	−61.5 (2)
C28—C17—C18—C19	179.91 (17)	C58—C47—C48—C49	−178.29 (17)
C22—C17—C18—C19	58.6 (2)	C52—C47—C48—C49	59.5 (2)
C16—C17—C18—C19	−68.2 (2)	C46—C47—C48—C49	−67.0 (2)
C28—C17—C18—C13	−44.32 (19)	C58—C47—C48—C43	−42.94 (18)
C22—C17—C18—C13	−165.60 (18)	C52—C47—C48—C43	−165.19 (17)
C16—C17—C18—C13	67.5 (2)	C46—C47—C48—C43	68.39 (19)
O3—C13—C18—C19	170.07 (18)	O8—C43—C48—C49	170.64 (19)
C12—C13—C18—C19	−79.5 (2)	C42—C43—C48—C49	−79.6 (2)
C14—C13—C18—C19	56.8 (3)	C44—C43—C48—C49	57.2 (3)
O3—C13—C18—C17	43.22 (18)	O8—C43—C48—C47	43.61 (17)
C12—C13—C18—C17	153.66 (17)	C42—C43—C48—C47	153.42 (16)
C14—C13—C18—C17	−70.1 (2)	C44—C43—C48—C47	−69.8 (2)
C17—C18—C19—C20	−56.9 (2)	C47—C48—C49—C50	−58.1 (2)
C13—C18—C19—C20	−178.49 (19)	C43—C48—C49—C50	−179.81 (19)
C18—C19—C20—C29	171.52 (17)	C48—C49—C50—C60	−70.7 (2)
C18—C19—C20—C30	−70.0 (2)	C48—C49—C50—C59	170.21 (19)
C18—C19—C20—C21	53.2 (2)	C48—C49—C50—C51	52.1 (2)
C29—C20—C21—C22	−170.98 (19)	C60—C50—C51—C52	71.6 (2)
C30—C20—C21—C22	70.1 (2)	C59—C50—C51—C52	−168.9 (2)
C19—C20—C21—C22	−53.3 (3)	C49—C50—C51—C52	−50.8 (3)
C28—C17—C22—C21	−166.31 (19)	C58—C47—C52—C51	−166.46 (19)
C18—C17—C22—C21	−54.9 (3)	C48—C47—C52—C51	−53.9 (2)
C16—C17—C22—C21	70.0 (2)	C46—C47—C52—C51	70.0 (2)
C20—C21—C22—C17	53.7 (3)	C50—C51—C52—C47	51.6 (3)
C15—C14—C27—O5	17.2 (3)	C45—C44—C57—O10	11.7 (3)
C13—C14—C27—O5	134.0 (2)	C43—C44—C57—O10	128.7 (2)
C8—C14—C27—O5	−104.4 (2)	C38—C44—C57—O10	−110.0 (2)
C13—O3—C28—O4	179.0 (2)	C43—O8—C58—O9	−177.56 (19)
C13—O3—C28—C17	−2.9 (2)	C43—O8—C58—C47	1.0 (2)
C22—C17—C28—O4	−32.7 (3)	C52—C47—C58—O9	−35.7 (3)
C18—C17—C28—O4	−151.5 (2)	C48—C47—C58—O9	−154.5 (2)
C16—C17—C28—O4	94.4 (3)	C46—C47—C58—O9	91.7 (3)
C22—C17—C28—O3	149.32 (19)	C52—C47—C58—O8	145.88 (18)
C18—C17—C28—O3	30.6 (2)	C48—C47—C58—O8	27.1 (2)
C16—C17—C28—O3	−83.6 (2)	C46—C47—C58—O8	−86.7 (2)

### Hydrogen-bond geometry (Å, º)

**Table:**

*D*—H···*A*	*D*—H	H···*A*	*D*···*A*	*D*—H···*A*
O2—H2···O4^i^	0.86 (3)	1.94 (3)	2.782 (2)	167 (3)
O7—H7···O6^ii^	0.90 (3)	1.91 (3)	2.803 (2)	172 (2)

Symmetry codes: (i) *x*−1/2, −*y*+1/2, −*z*; (ii) −*x*+2, *y*−1/2, −*z*+1/2.
